# Rapid Detection and Quick Characterization of African Swine Fever Virus Using the VolTRAX Automated Library Preparation Platform

**DOI:** 10.3390/v16050731

**Published:** 2024-05-05

**Authors:** Vivian O’Donnell, Jim L. Pierce, Oleg Osipenko, Lizhe Xu, Amy Berninger, Steven M. Lakin, Roger W. Barrette, Douglas P. Gladue, Bonto Faburay

**Affiliations:** 1 Plum Island Animal Disease Center, Animal and Plant Health Inspection Service, U.S. Department of Agriculture, Foreign Animal Disease Diagnostic Laboratory, National Veterinary Services Laboratories, Orient, NY 11957, USA; lizhe.xu@usda.gov (L.X.); roger.w.barrette@usda.gov (R.W.B.); bonto.faburay@usda.gov (B.F.); 2 Oak Ridge Institute for Science and Education (ORISE), Oak Ridge, TN 37830, USA; jim.l.pierce@usda.gov (J.L.P.); amy.berninger@usda.gov (A.B.); 3 National Bio- and Agro-Defense Facility, Animal and Plant Health Inspection Service, U.S. Department of Agriculture, Manhattan, KS 66502, USA; oleg.osipenko@usda.gov (O.O.); steven.lakin@usda.gov (S.M.L.); 4 National Bio- and Agro-Defense Facility, Agricultural Research Service, U.S. Department of Agriculture, Manhattan, KS 66502, USA; douglas.gladue@usda.gov; 5 Plum Island Animal Disease Center, Agricultural Research Service, U.S. Department of Agriculture, Orient, NY 11957, USA

**Keywords:** VolTRAX, African swine fever, next-generation sequencing, nanopore

## Abstract

African swine fever virus (ASFV) is the causative agent of a severe and highly contagious viral disease affecting domestic and wild swine. The current ASFV pandemic strain has a high mortality rate, severely impacting pig production and, for countries suffering outbreaks, preventing the export of their pig products for international trade. Early detection and diagnosis of ASFV is necessary to control new outbreaks before the disease spreads rapidly. One of the rate-limiting steps to identify ASFV by next-generation sequencing platforms is library preparation. Here, we investigated the capability of the Oxford Nanopore Technologies’ VolTRAX platform for automated DNA library preparation with downstream sequencing on Nanopore sequencing platforms as a proof-of-concept study to rapidly identify the strain of ASFV. Within minutes, DNA libraries prepared using VolTRAX generated near-full genome sequences of ASFV. Thus, our data highlight the use of the VolTRAX as a platform for automated library preparation, coupled with sequencing on the MinION Mk1C for field sequencing or GridION within a laboratory setting. These results suggest a proof-of-concept study that VolTRAX is an effective tool for library preparation that can be used for the rapid and real-time detection of ASFV.

## 1. Introduction

African swine fever (ASF) is a highly contagious hemorrhagic disease that causes serious economic losses to the swine industry due to the high mortality rate of domestic swine and the impact on international trade for countries that have ASF outbreaks. ASF is caused by ASFV, the only member of the *Asfivirus* genus within the *Asfarviridae* family, an enveloped and double-stranded DNA virus with a genome of approximately 170–190 kbp encoding 150–200 genes [1]. Historically, 24 genotypes have been described based on a partial sequence of the p72 (B646L) gene C-terminal region; however, recently it has been described that only six genotypes exist based on the protein coding sequence of p72 [2] and eight serogroups based on the CD2 (EP402R) gene [3].

ASF was described for the first time in Africa in 1921 [4] and remained in Africa with the exception of sporadic outbreaks of genotype I that were able to be controlled. However, the introduction of ASFV Genotype II into Georgia in 2007 subsequently spread into Russia and the European Union (EU) in 2014; then, in 2018, it entered China, which led to further spread across Asia. Recently, ASFV reached the Caribbean islands (2021) [5,6], the first reintroduction to the Western Hemisphere since its eradication in 1980 from the island of Hispaniola [7,8]. The expanding spread of ASF outbreaks raises concerns regarding its potential introduction into countries free from the virus. To quickly establish control of the disease, it is necessary to implement rapid detection and surveillance programs, the culling of infected animals, establishment of control zones, movement restrictions, the tracing of pig movement and possible contacts, and the depopulation of affected premises. At present, there is no treatment or world-wide available vaccine for ASF, except for Vietnam where there are two commercially available live-attenuated vaccines for ASF. Both are live-attenuated vaccines with genetically modified deletions [9,10,11]. As ASF vaccines become more available, vaccine matching would have to be performed and would rely on rapid whole genome sequencing of ASFV to ensure a vaccine would be effective and not potentially cause recombination events [12]. At this time, vaccination is strictly prohibited in the EU and other countries [1]; therefore, disease control is currently dependent on timely and reliable diagnosis, along with well-planned surveillance to control the spread of the disease.

Previously, we described the use of Nanopore technology and a companion analysis ASF-FAST software, to allow real-time detection of the ASFV genome sequence [13]. Our results demonstrated that the use of Nanopore sequencing technology could be applied for sequence-based diagnosis, effectively supporting emergency management in the event of an outbreak of the disease.

In this work, we evaluated the VolTRAX platform (Oxford Nanopore Technologies, Oxford, UK) as a proof-of-concept portable device to automate the preparation of end-to-end libraries for sequencing. VolTRAX has been developed by Oxford Nanopore Technologies (ONT), as a small programmable device designed to convert DNA out of a biological sample into a library ready for sequencing into a nanopore device, such as MinION Mk1C or GridION. The libraries are automatically prepared by applying a charge, using a rapid chemistry kit and predefined protocols. With VolTRAX, only a few minutes of hands-on work is necessary, and only 45 min was necessary for the completion of the next-generation sequencing libraries, which can be used for downstream sequencing. This type of methodology can be of great interest and application as a point-of-care (POC) device, similar to conventional PCR, for the rapid detection of ASFV in the field. However, POC PCR relies on the detection of a small fraction of the ASFV genome covering the p72 region (400 bp) and would not provide meaningful functional information on the ASFV isolate, such as the genotype [2] or the information necessary to characterize the virus. VolTRAX and Nanopore sequencing allow the whole genome of ASFV to be used for field diagnosis, providing in-depth information about the ASF outbreak independent from extensive sample preparation protocols needed for other traditional NGS platforms. This work demonstrates for the first time a proof of concept of the efficiency of the VolTRAX platform for the automated preparation of reliable and consistent libraries from whole-blood samples and Nanopore sequencing, as a method for the rapid and real-time detection of ASFV, which can be implemented in a non-laboratory environment with low or no infrastructure.

## 2. Materials and Methods

### 2.1. Porcine Whole-Blood Samples for the Detection of ASFV

Blood samples were obtained from pigs experimentally infected by intramuscular administration with 10^5^ TCID50/mL of ASFV Georgia 2007/1, 10^5.4^ TCDI50/mL of ASF Lisbon/60, or 10^4^ HAD50/mL of ASF Dominican Republic-21 (DR-21). ASF Georgia 2007/1, Lisbon/60, and DR-21 were selected as the most representative strains for genotypes I and II. Blood samples (EDTA stabilized) were collected from the following: eleven ASFV Georgia 2007/1 infected pigs, sampled at 6–9 days post-inoculation (d.p.i.); eight ASFV Lisbon/60 infected pigs, sampled at 5–7 d.p.i.; and one ASF DR-21 infected pig, sampled at 12 d.p.i. The blood samples were taken during the acute phase of ASFV infection, when clinical signs of the disease were observed. The samples were processed immediately following collection (fresh blood samples) or stored at −70 °C (frozen blood sample). All samples from experimentally infected pigs were provided by the Foreign Animal Disease Diagnosticians (FADD) Training Course, USDA-NVSL-FADDL. As negative controls, three negative blood samples from domestic pigs were included.

### 2.2. ASFV Strains Used for Evaluation

Six ASFV strains, Georgia 2007/1, Killean III, Kimakia-64, Malawi Lil-20/1, DR/2021, and Pretoria-4/1, provided by the U.S. Department of Agriculture, National Veterinary Services Laboratories’ Foreign Animal Disease Diagnostic Laboratory (USDA-NVSL-FADDL), were used to prepare viral stocks in primary swine macrophages, as described elsewhere [14]. Briefly, primary cultures of swine macrophages were prepared from swine blood, and the macrophages were plated at 2 × 107 cells/well on T25 Primaria flasks. The flasks were inoculated with 100 uL of each ASF viral strain and incubated at 37 °C under 5% CO2 for 5 days. The presence of virus was assessed by hemadsorption (HA); the plates were frozen at −70 °C, clarified by centrifugation at 4000 rpm/20 min at 4 °C, aliquoted, and stored at −70 °C for further use. The viruses were spiked 1:1 into blood from naïve animals to ensure that libraries were prepared using the same matrix, as a diagnostic fresh blood sample.

### 2.3. ASFV Nucleic Acid Extraction

Total nucleic acid extractions were performed using the Taco-Mini device (GeneReach, Taichung City 407, Taiwan). The Taco-Mini is an automated magnetic bead-based total nucleic acid extraction platform that allows extraction of 8 samples at a time [15], with reagents stored at room temperature (RT). Triplicate extractions, when evaluating the consistency of library preparation with VolTRAX, or single extractions, when testing the VolTRAX as a tool for rapid library preparation, were performed. Once the extractions were completed, the nucleic acids were transferred to LoBind tubes and processed immediately. The aliquots of all nucleic acid extracts were quantified using the Qubit dsDNA high sensitivity kit (ThermoFisher, Waltham, MA, USA) and used for library preparation and for real-time PCR.

### 2.4. Detection of ASFV Using Real-Time PCR

Detection of ASFV in all samples was performed using specific primers and probes to amplify the gene sequence encoding the major ASFV p72 capsid protein, as a modification of Zsak et al. [16]. Each 25 µL reaction mixture contained 2.5 µL of nucleic acid template, 1.25 µL of primer–probe mix (forward: CTT Cgg CgA gCg CTT TAT CAC, reverse: ggA AAT TCA TTC ACC AAA TCC TT, probe: FAM—CgA TgC AAg CTT TAT—MGB/NFQ), 6.25 µL of enzyme mix (TaqMan^®^ Fast Virus 1-Step Master Mix, ThermoFisher), and 15 µL of nuclease-free water. Real-time PCR (rt-PCR) was performed using an rt-PCR system (ABI7500, ThermoFisher). The cycling conditions consisted of denaturation at 95 °C for 20 s (1 cycle), 45 cycles of amplification at 95 °C for 10 s, and then 60 °C for 30 s in standard run mode. Samples with a threshold cycle (Ct) value equal to or less than 40 were considered positive, which is consistent with the NVSL FADDL testing algorithm for ASFV [17].

### 2.5. VolTRAX DNA Library Preparation and Sequencing

VolTRAX V2002b (ONT) was used for automated library preparation, using VolTRAX V2 cartridges (VCT-V2002B, ONT). For all experiments, VolTRAX sequencing reagents were added to the cartridge, and a total of 9.5 µL of the extracted nucleic acid was used as input for the preparation of the libraries. Even when the VolTRAX kit suggested an input of 50 ng/ul of total nucleic acid, no normalization was performed to adjust the concentrations, due to limitations in the field, such as determining the concentration of DNA. For single-plex libraries, the VSK-VSK004 kit (ONT), which is based on rapid sequencing chemistry, using a tagmentation-based technology and bead clean up step, was used. Evaluation of the multiplex library preparation was performed with the VSK-VMK004 kit (ONT). The multiplex kit uses rapid chemistry, with the capacity to multiplex up to ten samples on one VolTRAX cartridge (VCT-V2002B, ONT), and a bead-based clean up step after barcoding. Both libraries, single-plex or multiplex, were prepared following a series of steps programmed by the VolTRAX V2b software. The libraries were loaded onto R9.4.1 flow cells (FLO-MIN106), on Nanopore next-generation sequencers, MinION Mk1C (ONT)or GridION (ONT), and were run for 48–72 h. MinION Mk1C and GridION devices have no differences in individual flow cell functionality; therefore, the samples were run as indistinguishable on any platform. All sequencing runs on both platforms were set up for high-accuracy base calling with a q score of 20, with a minimum q score of 9, as the default for the MinKNOW software version 23.11.03.

### 2.6. Bioinformatic

Raw reads wre produced on an instrument with MinKNOW software, which controls all nanopore devices for base calling in real time. Data were initially filtered by the MinKNOW software’s default parameters, which include an algorithm to convert electrical signals to base pair, and Guppy, a high accuracy model for base calling, which is integrated within MinKNOW (https://nanoporetech.com/platform/technology/basecalling) accessed on 17 April 2024. Reads were aligned, using Minimap2 [18], to a representative set of complete ASFV reference genomes obtained from the GenBank database as of January, 2023 (Supplementary Table S1). The total number of reads aligning and the genome coverage metrics were calculated. The genome that maximized the average alignment score across all aligned base pairs was used to infer the genotype and closest strain for each sample. The average alignment score measures both the genomic identity and coverage and was calculated using the following scores: match = 2, mismatch = −4, indel = −2, with an optional affine gap penalty, which was not used in this analysis [19].

## 3. Results and Discussion

### 3.1. Evaluation of VolTRAX to Prepare Reliable and Consistent Libraries

**Figure 1:**
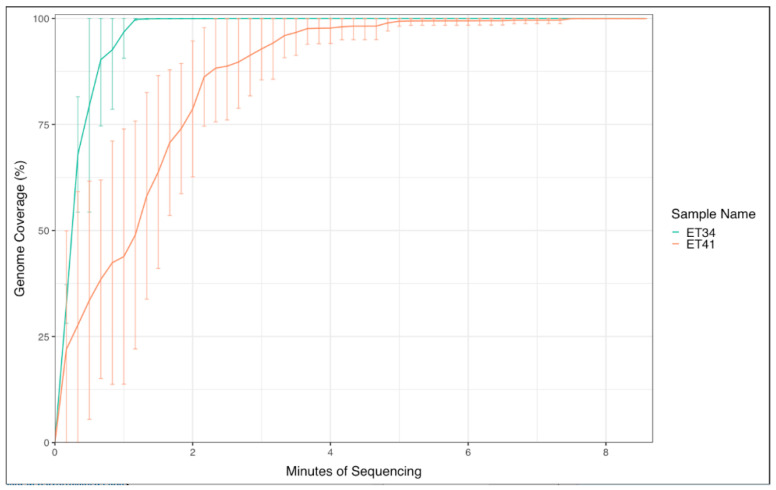
ASFV time-course sequencing using fresh whole-blood samples from pigs experimentally infected with ASFV Georgia 2007/1 (ET41) or ASFV Lisbon/60 (ET34). Each sample was extracted in triplicate, and libraries were prepared from each replicate. Shown is the % of the coverage of the ASFV genome. Data are plotted with 95th percentiles for all three replicates for each of the two pigs tested.

To evaluate the capability of the VolTRAX platform to provide reliable and consistent end-to-end libraries to detect the ASFV genome, fresh whole-blood samples from pigs experimentally infected with ASFV were used. For this experiment, three independent extractions from whole-blood samples from the same pig infected with ASFV Georgia 2007/1, taken at 9 d.p.i., and three independent extractions from whole-blood samples from a pig infected with ASFV Lisbon/60, taken at 7 d.p.i., were processed after collection. The DNA concentrations were determined to have an estimation for the range of input DNA (Table 1). However, due to the limitations that may be present with the use of the VolTRAX in the field, such as not being able to perform additional DNA analysis, no further normalization steps were performed, with the idea being to mimic how samples will be taken in the field. All libraries that were obtained from each of the three replicates per animal showed concentrations ranging from 11.9 ng/uL to 25.2 ng/uL for animals infected with Georgia 2007/1 and 3.28 ng/uL to 6.56 ng/uL for animals infected with Lisbon/60 (Table 1). The libraries were then individually sequenced on Nanopore next-generation sequencers to determine the minimal time required for the ASFV genome to be sequenced and the consistency between different repeats from the same sample to resolve the ASF genome. As early as 2 min for Georgia 2007/1 and 4 min for Lisbon/60 after sequence initiation, over 90% of the ASFV genome was resolved with enough depth to determine the ASF isolate, and there was similar coverage at the end of the runs between the three replicates per sample (coverage over 99.9% for all replicates, Figure 1, Table 1). These results were consistent with our previously published work with manual library preparation [13].

**Figure 2:**
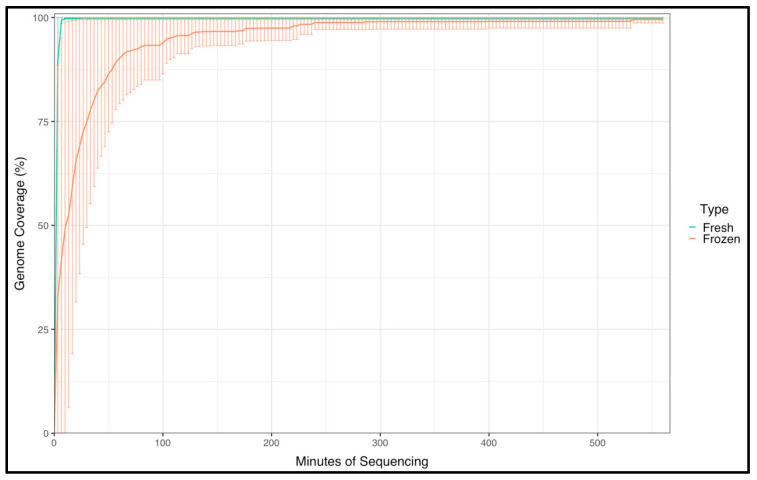
ASFV frozen whole-blood sample and fresh whole-blood sample from a pig experimentally infected with ASFV Georgia 2007/1 were processed for library preparation with VolTRAX and sequenced on Nanopore next-generation sequencers to evaluate the consistency of library preparation when frozen samples are used. Each blood sample, fresh or frozen, was extracted in triplicate, and libraries were prepared from each replicate. Shown is the % of the coverage of the ASFV genome for both animals. Data are plotted with 95th percentiles for all three replicates for each of the two samples.

Additionally, frozen blood samples were evaluated to determine whether samples that might not immediately be processed in the field were viable using this automated system. Similar to the fresh blood samples, three independent extractions from a whole-blood sample, fresh or frozen, from the same pig infected with ASFV Georgia 2007/1 (6 d.p.i.) were evaluated. As observed previously, the libraries were consistent between the repeats for the same sample, with a coverage between 98% and 99% (Table 2). The frozen sample repeats required a longer time for resolution of the almost complete genome, taking at least 100 min to resolve 85% of the ASFV genome (Figure 2). Our results demonstrate the consistency on the coverage of the libraries prepared, mostly due to the simple workflow and the precision of the fluid handling on the VolTRAX platform, confirming that the device was capable of performing ASFV genome sequencing and resolving the ASFV genome with a coverage above 98% when fresh or frozen samples were evaluated, and enough depth was obtained to characterize the ASF strain used.

### 3.2. Detection of ASFV from Fresh Whole-Blood Samples in Real Time

**Figure 3:**
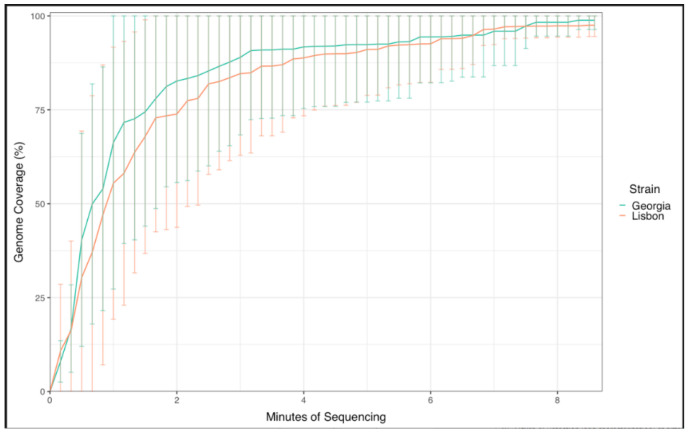
ASFV time-course full-genome sequencing using fresh whole-blood samples from pigs experimentally infected with ASFV Georgia 2007/1 (n = 5) or ASFV Lisbon/60 (n = 5). Shown is the % of the coverage of the ASFV genome for all animals per strain. Data are plotted with 95th percentiles for all five pigs per strain used for inoculation.

To evaluate the VolTRAX as a portable device to bring into the field as a tool for the rapid detection of ASFV, where the location of ASF outbreaks may have limited access to adequately equipped laboratories, several fresh blood samples from pigs experimentally infected with ASFV Georgia 2007/1 (genotype II) or Lisbon/60 (genotype I) were processed. Blood samples were taken during the acute phase of ASFV infection, when clinical signs of the disease were observed, as this will be the sample of preference to sequence first in an outbreak investigation. Extractions of nucleic acids out of fresh whole-blood EDTA samples from pigs experimentally infected with ASFV were performed on the Taco device, and concentrations were determined by quantitating on a Qubit device to have a reference for the starting material (Table 3). As previously described, no normalization steps were performed, in order to simulate samples processed in the field. The samples used for this experiment showed Ct values between 17.45 and 19.75 (Table 3), which ensured obtaining a good quality sequence for ASFV, as described by Shi et al. [20]. Concentrations obtained from the extractions ranged from 21 to 72 ng/µL (Table 3). Even when some samples did not have the preferred concentration of nucleic acid indicated by the manufacturer as 50 ng/uL, we continued to process them to evaluate the capabilities of the VolTRAX platform for implementation under field conditions for library preparation. Once the libraries were prepared, the concentrations were determined, and the libraries were individually loaded onto Nanopore flow cells. The results showed a depth between 3 X and 29 X, with a coverage of more than 94% for all the samples sequenced. All the samples were resolved to at least 80% of the genome within a few minutes after the runs started (~7 min, Figure 3). Three negative blood samples obtained from domestic pigs were also included as negative controls. The three samples tested negative for ASF by rt-PCR and resulted in no reads detected that aligned with ASF, as shown in Table 3. Overall, the VolTRAX platform for the rapid generation of NGS libraries performed very well when fresh blood samples were evaluated and allowed resolution of nearly complete ASFV genomes within minutes after runs were started, suggesting that this device would be promising under field conditions for the rapid detection and identification of ASFV genomes.

### 3.3. Evaluation of VolTRAX to Detect ASFV in Frozen Blood Samples

The ideal use of this platform is to run samples as they are collected in the field; however, we evaluated a limited number of whole-blood EDTA frozen samples, as there will be some cases where samples cannot be immediately processed. Extractions of nucleic acids out of samples were performed on Taco from pigs experimentally infected (6–9 dpi) with ASFV Georgia 2007/1 (genotype II), Lisbon/60 (genotype I), or DR-21 (genotype II); the samples used for this experiment are indicated in Table 4. Concentrations obtained from the extractions ranged from 7 to 95 ng/µL.

The libraries generated with VolTRAX and sequenced on Nanopore next-generation sequencers showed a depth between 1.58X and 6.5X, with a coverage between 75.1% (one sample) and 99.95%. The data, even with a limited number of samples, indicate that the VolTRAX can generate libraries that are consistent and reliable for sequencing to obtain at least 75.1% of coverage and allow the characterization of the virus by genome analysis.

### 3.4. Detection and Characterization of Different ASFV Genotypes with VolTRAX

**Figure 4:**
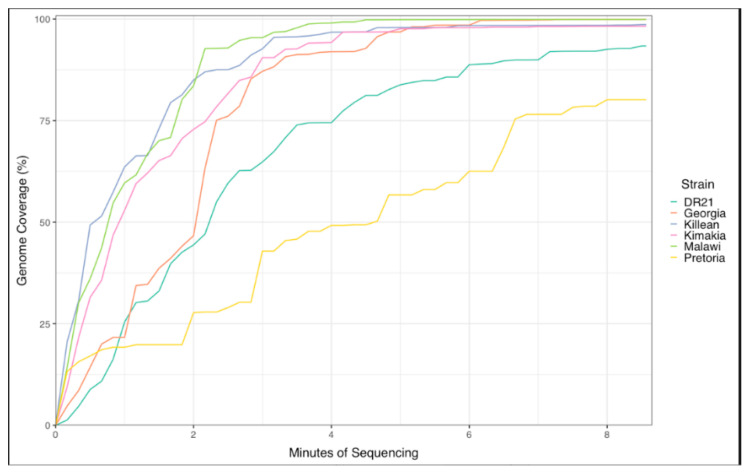
Capability of the VolTRAX to prepare libraries out of different ASFV isolates and their characterization by Nanopore sequencing. Libraries prepared independently from six different ASFV viral stocks were loaded individually onto the primed Nanopore flow cells.

We evaluated the capacity of the VolTRAX to prepare libraries out of genetically distinct ASFV isolates (Georgia 2007/1, Killean III, Kimakia-64, Malawi Lil-20/1, DR-21, and Pretoria-4/1). ASFV strains were obtained from the USDA FADDL Biorepository; stocks that were grown on primary porcine macrophage cell cultures were used to spike blood from naïve animals to mimic a fresh diagnostic sample. Libraries prepared with VolTRAX were run on Nanopore next-generation sequencers. For all the isolates, more than 70% of the genome was resolved between 2 and 6 min after the runs were started. Most of the samples were resolved to greater than 93% of the genome, but the stock prepared out of Pretoria-4/1 only had 80% of the genome resolved. Even though the coverage only reached 80% (one sample, Pretoria-4/1) and 93–99% for other samples, the sequences obtained were sufficient to easily characterize each of the isolates by their corresponding genotype within 6 and 8 min (Figure 4, Table 5).

### 3.5. Evaluation of the Multiplex Capability of the VolTRAX Platform

VolTRAX has the capability to multiplex up to 10 samples per cartridge when combined with the multiplex kit; however, the performance seems to decrease with the additional multiplexing of samples. Therefore, to evaluate how the kit performs when multiplexing and whether samples are equally represented when multiplexed, first, we selected one fresh whole-blood sample from an experimentally infected pig with ASFV Georgia 2007/1 out of the samples run on the single-plex format previously. Libraries were prepared to the limit of multiplexing available on the VolTRAX device; so, 10 replicates out of the same sample were loaded to test the multiplex ability of the VolTRAX cartridge when using the multiplex kit. The barcoded and pooled library resulted in a concentration of 10.7 ng/µL, which was loaded and sequenced on a Nanopore next-generation sequencer. Even when the same sample was equally loaded in the cartridge, the results showed a range from 5% to 37% coverage with a very low average depth (0.03 X to 0.4 X), which indicates that the samples are not equally represented when multiplexed libraries are loaded on Nanopore platforms. Even when the objective of the Multiplex kit is to achieve equal representation of each sample and barcode when multiplexing, several factors such as the experimental conditions, the efficiency of transposition, and the subsequent steps, as described by the manufacturer, can have an impact on the final distribution of the barcodes, as seen in this study, when using replicates out of the same sample. To further evaluate the multiplex kit, we selected 10 fresh whole-blood EDTA samples, some of them already sequenced as single plex, and the experiment was run in duplicate. Similarly, samples were not fully resolved, with a coverage ranging from 1% to 44–63% of the genome and a low average depth (0.03 X to 0.24 X). To allow an increase in the representation of each sample when multiplexing, we further evaluated the VolTRAX multiplexing with just four samples. For this experiment, each sample was loaded in duplicate using the same barcode per duplicate to increase the final representation of the sample to be sequenced. The pooled libraries resulted in 9.52 ng/µL (replicate 1) and 9.72 ng/µL (replicate 2). For most of the samples, more than 50% of the genome was resolved between 2 and 6 min after the runs were started with a coverage between 60 and 99.9% of the genome, with good consistency on the final results between both replicates (Table 6). Even though the sequences obtained when multiplexing resulted in a low average depth and were not fully resolved, it was enough coverage to detect ASFV and characterize the samples by their corresponding genotype. Even when the Multiplex kit may not have performed perfectly when limiting the capacity of ten samples, the data we obtained with the reduced number of samples, may help for future studies for ASFV automated library preparation when multiplexing samples. While these results were promising, additional development and further evaluation of the multiplex capabilities of the VolTRAX need to be considered. Our results were in agreement, as described by ONT for the VolTRAX multiplex kit, which has not yet been fully optimized for multiplexing at this time.

## 4. Conclusions

In this report, we evaluated as a proof of concept for ASF that VolTRAX has the capability to be used as a point-of-care device in the field in outbreak scenarios. The small footprint device requires minimal hands-on activity, utilizes field-stable reagents, and performs well under field conditions for fresh or frozen ASFV for the detection and characterization of ASFV genomes. Further evaluation under field conditions could be performed to evaluate this system for use in the field for rapid identification and diagnostics to determine the necessary number of animals that need to be tested in order to have a reliable system to quickly identify strains of ASFV that are causing outbreaks. It will also be important to evaluate the minimal training required for onsite use. The VolTRAX system is the most compact and user-friendly system for this use. In this report, we presented laboratory data that suggest that this system could have value in certain outbreak situations, where having the ability to fully sequence ASFV could be necessary for both a rapid diagnostic to identify the strain of ASFV during an initial outbreak and for potential vaccine matching, as vaccines become more widely available. In addition, this type of onsite sequencing could be used to quickly identify new emerging strains of ASFV that are causing outbreaks. Having this information quickly and accurately is necessary for the rapid response and control of isolated outbreaks. However, this will have to be first evaluated under field conditions during outbreaks to determine whether this is a feasible methodology for future use. Further evaluation is also required to optimize the multiplexing of samples, which could allow a larger number of samples to be sequenced at the same time, reducing costs, and increasing the number of genomes rapidly sequenced during an initial outbreak investigation.

## Figures and Tables

**Table 1:** Evaluation of consistency on libraries prepared with VolTRAX and sequenced on Nanopore next-generation sequencers using fresh blood samples from a pig experimentally infected with ASFV Georgia 2007/1 (9 d.p.i.) or a pig experimentally infected with Lisbon/60 (7 d.p.i.). Each sample was extracted in triplicate (replicates 1, 2, and 3). Each extract was individually used as input to prepare the libraries. Input DNA and concentration of library are expressed in ng/µL.

ASFV Strain/ Genotype	Replicate	ASF rt-PCR (Ct)	Input DNA (ng/µL)	Library (ng/µL)	Depth	Coverage
Georgia 2007/1/II	1	19.7	44.5	11.9	5.8 X	99.95%
Georgia 2007/1/II	2	20.32	66.7	17.3	6.4 X	99.97%
Georgia 2007/1/II	3	20.35	90	25.2	10.6 X	99.99%
Lisbon/60/I	1	17.49	21.9	3.28	34.75 X	99.99%
Lisbon/60/I	2	17.45	27.4	6.56	54.8 X	99.99%
Lisbon/60/I	3	17.93	21.2	6.38	31.14 X	99.99%

**Table 2:** Evaluation of the consistency of libraries obtained with VolTRAX and sequenced on Nanopore next-generation sequencers when using frozen blood samples as compared with fresh blood samples from a pig experimentally infected with ASFV Georgia 2007/1 (6 d.p.i.). Each sample, fresh and frozen, was extracted in triplicate (replicates 1, 2, and 3), and each extract was used as input to prepare the libraries. Input DNA and concentration of library are expressed in ng/µL.

ASFV Strain	Replicate	Sample Type	ASF rt-PCR (Ct)	Input DNA (ng/µL)	Library (ng/µL)	Depth	Coverage
Georgia 2007/1	1	Fresh	19.94	43	6.72	6.7 X	99.47%
Georgia 2007/1	2	Fresh	19.78	28.8	7.58	4.33 X	98.72%
Georgia 2007/1	3	Fresh	19.67	45	7.76	4.03 X	99.88%
Georgia 2007/1	1	Frozen	19.06	74	23.4	5.4 X	99.96%
Georgia 2007/1	2	Frozen	19.6	76	24	4.1 X	97.96%
Georgia 2007/1	3	Frozen	18.35	76.6	19.7	6.5 X	99.95%

**Table 3:** Summary of ASFV fresh whole-blood samples to evaluate the competency of the VolTRAX for library preparation along with Nanopore next-generation sequencing as a rapid tool. A. Samples correspond to five pigs experimentally infected with ASFV Georgia/2007 and five pigs experimentally infected with ASFV Lisbon/60. B. Whole-blood samples corresponding to three domestic pigs used as negative controls. Input DNA and concentration of library are expressed in ng/µL.

**A. ASF experimentally infected pigs.**						
**ASFV Strain**	**d.p.i.**	**ASF** **rt-PCR (Ct)**	**Input DNA (ng/µL)**	**Library** **(ng/µL)**	**Depth**	**Coverage**
Georgia 2007/1	7	18.24	36.8	11.8	29.8 X	99.99%
Georgia 2007/1	6	18.44	72.8	27.8	13 X	99.99%
Georgia 2007/1	6	19.31	26	3.64	3.01 X	95.61%
Georgia 2007/1	9	19.05	58	10.4	22.88 X	99.99%
Georgia 2007/1	6	19.63	21.9	6.28	7.72 X	99.93%
Lisbon/60	9	17.45	39.6	21.8	16 X	99.99%
Lisbon/60	9	19.67	69.2	19.1	3.17 X	94.85%
Lisbon/60	7	19.66	22.6	9.22	13.67 X	99.99%
Lisbon/60	6	17.83	47.5	9.94	2.4 X	94.61%
Lisbon/60	6	19.75	45.8	20.6	6.4 X	98.18%
**B. Negative controls.**						
**Negative** **Control**		**ASF** **rt-PCR (Ct)**	**Input DNA (ng/µL)**	**Library** **(ng/µL)**	**Depth**	**Coverage**
Negative # 1		Negative	51.2	6.56	0 X	0%
Negative # 2		Negative	54	6.80	0 X	0%
Negative # 3		Negative	17.9	2.5	0 X	0%

**Table 4:** Summary of ASFV frozen whole-blood samples to evaluate the competency of the VolTRAX for library preparation along with Nanopore next-generation sequencing. Samples correspond to seven pigs experimentally infected with ASFV Georgia/2007, ASFV Lisbon/60, or ASFV DR-21.

ASFV Strain	d.p.i.	ASF rt-PCR (Ct)	Input DNA (ng/µL)	Library (ng/µL)	Depth	Coverage
Georgia 2007/1	7	19.6	19	17.2	6.5 X	99.95%
Georgia 2007/1	7	21.15	77.6	25.4	3.05 X	89.18%
Georgia 2007/1	6	19.6	100	23	1.58 X	75.1%
Georgia 2007/1	6	19	20	4	1.92 X	87.08%
Lisbon/60	6	19.79	47.5	9.94	1.95 X	82.17%
Lisbon/60	7	20.55	94.7	26.6	2.4 X	94.6%
DR-21	12	19	6.64	1.77	1.7 X	97.59%

**Table 5:** Summary of the ASFV isolates run to evaluate VolTRAX and sequencing on Nanopore next-generation sequencers using virus stocks that were produced in primary swine macrophages. Input DNA and concentration of library are expressed in ng/µL.

ASFV Strain	ASF rt-PCR(Ct)	Input DNA (ng/µL)	Library (ng/µL)	Depth	Coverage
Georgia/2007	20.7	14	3.92	5.8 X	99.92%
DR-21	24	12.1	2.64	3.2 X	93.39%
Kimakia-64	19.98	6.18	1.16	8.6 X	98.23%
Killean III	19.95	6.16	1.19	9.2 X	98.65%
Pretoria-4/1	19	12.2	2.08	1.3 X	80.16%
Malawi Lil-20/1	19.9	16.8	2.76	11 X	99.89%

**Table 6:** Summary of the ASFV samples run to evaluate the multiplexing capabilities on VolTRAX and the sequencing on Nanopore next-generation sequencers. To increase the final representation of each sample to be sequenced, a set of four fresh whole-blood samples from four different pigs infected with ASF Georgia 2007/1 were evaluated, loading each sample in duplicate with the same barcode. Experiments were run in duplicate.

ASFV Strain	Sample	d.p.i.	Sample Type	ASF	Replicate 1		Replicate 2	
				rt-PCR (Ct)	Depth	Coverage	Depth	Coverage
Georgia 2007/1	1	7	Fresh Blood	18.20	8.5 X	99.9%	4.45 X	99.88%
Georgia 2007/1	2	6	Fresh Blood	19.02	1.5 X	72.91%	0.42 X	38.46%
Georgia 2007/1	3	6	Fresh Blood	20.05	1.07 X	66.16%	0.77 X	60.61%
Georgia 2007/1	4	9	Fresh Blood	19	3.66 X	96.69%	1.75 X	81.91%

## Data Availability

All data are included in the manuscript. All ASFV reference genomes were obtained from GenBank, and the GenBank accession number of each sequence has been provided in Supplementary Table S1.

## Supplementary Materials

The following supporting information can be downloaded at: https://www.mdpi.com/article/10.3390/v16050731/s1, Supplementary Table S1: ASFV reference genomes.
